# Detection of the V1016G mutation in the voltage-gated sodium channel gene of *Aedes aegypti* (Diptera: Culicidae) by allele-specific PCR assay, and its distribution and effect on deltamethrin resistance in Thailand

**DOI:** 10.1186/1756-3305-6-253

**Published:** 2013-08-30

**Authors:** Steven A Stenhouse, Suriya Plernsub, Jintana Yanola, Nongkran Lumjuan, Anchalee Dantrakool, Wej Choochote, Pradya Somboon

**Affiliations:** 1 Department of Parasitology, Faculty of Medicine, Chiang Mai University, Chiang Mai 50200, Thailand; 2 Department of Medical Technology, Faculty of Associated Medical Sciences, Chiang Mai University, Chiang Mai 50200, Thailand; 3 Research Institute for Health Sciences, Chiang Mai University, Chiang Mai 50200, Thailand

**Keywords:** *Aedes aegypti*, *Kdr*, Pyrethroid, Deltamethrin resistance, AS-PCR, Thailand

## Abstract

**Background:**

Resistance to pyrethroid insecticides is widespread among populations of *Aedes aegypti*, the main vector for the dengue virus. Several different point mutations within the voltage-gated sodium channel (VGSC) gene contribute to such resistance. A mutation at position 1016 in domain II, segment 6 of the VGSC gene in *Ae. aegypti* leads to a valine to glycine substitution (V1016G) that confers resistance to deltamethrin.

**Methods:**

This study developed and utilized an allele-specific PCR (AS-PCR) assay that could be used to detect the V1016G mutation. The assay was validated against a number of sequenced DNA samples of known genotype and was determined to be in complete agreement. Larvae and pupae were collected from various localities throughout Thailand. Samples were reared to adulthood and their resistance status against deltamethrin was determined by standard WHO susceptibility bioassays. Deltamethrin-resistant and susceptible insects were then genotyped for the V1016G mutation. Additionally, some samples were genotyped for a second mutation at position 1534 in domain III (F1534C) which is also known to confer pyrethroid resistance.

**Results:**

The bioassay results revealed an overall mortality of 77.6%. Homozygous 1016G individuals survived at higher rates than either heterozygous or wild-type (1016 V) mosquitoes. The 1016G mutation was significantly and positively associated with deltamethrin resistance and was widely distributed throughout Thailand. Interestingly, wild-type 1016 V mosquitoes tested were homozygous for the 1534C mutation, and all heterozygous mosquitoes were also heterozygous for 1534C. Mutant homozygous (G/G) mosquitoes expressed the wild-type (F/F) at position 1534. However, the presence of the 1534C mutation was not associated with deltamethrin resistance.

**Conclusions:**

Our bioassay results indicate that all populations sampled display some degree of resistance to deltamethrin. Homozygous 1016G mosquitoes were far likelier to survive such exposure. However, resistance in some populations cannot be explained due to *kdr* mutations and indicates that other resistance mechanisms are operating. The presence of this mutation alone does not fully explain the resistance phenotype we see among Thai *Ae. aegypti* populations.

## Background

*Aedes aegypti* is an important disease vector and nuisance throughout its range. In Thailand, as in many other regions, the species is incriminated as the major vector for dengue virus. Dengue fever, as well as its hemorrhagic manifestations, presents major public health problems in Thailand [1] and millions of people are at continuous risk of this disease. Currently no vaccines or specific anti-viral medications are available. In the event of an outbreak, disease control efforts must resort to vector control. Reducing vector populations below thresholds capable of sustaining viral transmission requires the heavy use of space sprays of insecticides, usually pyrethroids. These insecticides are also widely used outside of an outbreak control context, in that they are used for ongoing, seasonal control efforts as well as being used in numerous households for personal protection against mosquitoes. Pyrethroid compounds are thus the primary insecticides used for the control of *Aedes* in Thailand. However, a number of reports from throughout the country show widespread and varying resistance to a variety of insecticides, including DDT, organophosphate compounds and pyrethroids [1-4].

Resistance in *Ae. aegypti*, as well as in other vector and pest species, may arise through two major mechanisms. The first mechanism consists of metabolic or enzymatic resistance. In this case resistance is achieved through the up-regulation or constitutive overproduction of detoxifying enzymes. They work by rapidly metabolizing and detoxifying the insecticide or by sequestration, therefore inhibiting or preventing the insecticide from binding its target site [5]. The second mechanism is knockdown resistance or *kdr*, which is resistance resulting from insecticide selection that is not overcome by metabolic inhibitors, such as piperonyl butoxide (PBO) [6]. This frequently consists of single point mutations within the genes coding for proteins that are targeted by insecticide compounds. Pyrethroid insecticides work by binding to voltage-gated sodium channels (VGSC) of neurons. They bind preferentially to open channels. Bound sodium channels then remain in the open, activated state which leads to repetitive nerve firing, which in turn leads to a loss of control and uncontrolled activity. The target insect experiences convulsions and is unable to maintain normal flight behavior [7]. However if certain point mutations within the VGSC gene are present, the resulting amino acid transversion may greatly decrease the sensitivity of the sodium channel to pyrethroid binding. It may also alter the conformation of the sodium channel to an extent that it remains closed and inactivated.

In Thailand, two common *kdr* mutations within the *Ae. aegypti* VGSC gene are known to be involved in pyrethroid resistance. A phenylalanine to cysteine substitution at position 1534 within the third domain of the VGSC (F1534C) is associated with resistance to permethrin. It has been previously shown to be widely distributed throughout Thailand [8]. Other studies have indicated that this mutation is widely distributed, having since been detected within the Caribbean [9], and in Vietnam [10]. A second mutation, involving a valine to glycine transversion in domain II (V1016G) is associated with resistance to the type II pyrethroid, deltamethrin. At present it appears to be restricted to Southeast Asia, including Thailand [11,12], Indonesia [13], Vietnam [10] and Taiwan [14]. The 1016G allele frequency was found to be 0.23 in a previous study [11]. A similar mutation at the same position (V1016I) occurs among *Ae. aegypti* populations from Latin America [15]. Additionally, *Ae. aegypti* from Thailand are known to express various enzymatic resistance mechanisms. Increased expression of mixed function oxidases relative to a susceptible strain has also been seen in various pyrethroid-resistant populations originating from Thailand [16,17]. Such metabolic mechanisms can contribute to resistance along with *kdr* mutations.

A number of PCR-based techniques exist for detecting this and other nucleotide polymorphisms in *Ae. aegypti*. For the V1016G mutation, an assay has been developed, but it is optimized for use in a real time PCR machine, although amplified products could be determined on an agarose gel [15]. Another technique recently developed utilizes a heated oligotide ligation assay for detection [11]. Although requiring only a thermal cycler, that assay also requires additional reagents that can contribute to increased costs. The assay we utilized here is simpler although genotyping results can only be determined by gel electrophoresis. The purpose of this study was to develop a simple allele-specific PCR-based assay (AS-PCR) to detect the V1016G mutation and then determine its role in deltamethrin resistance in Thailand. Such information is useful to vector control operations in determining the effects and distribution of one of the major mechanisms underpinning deltamethrin resistance.

## Methods

### Larval collection and rearing

**Figure 1:**
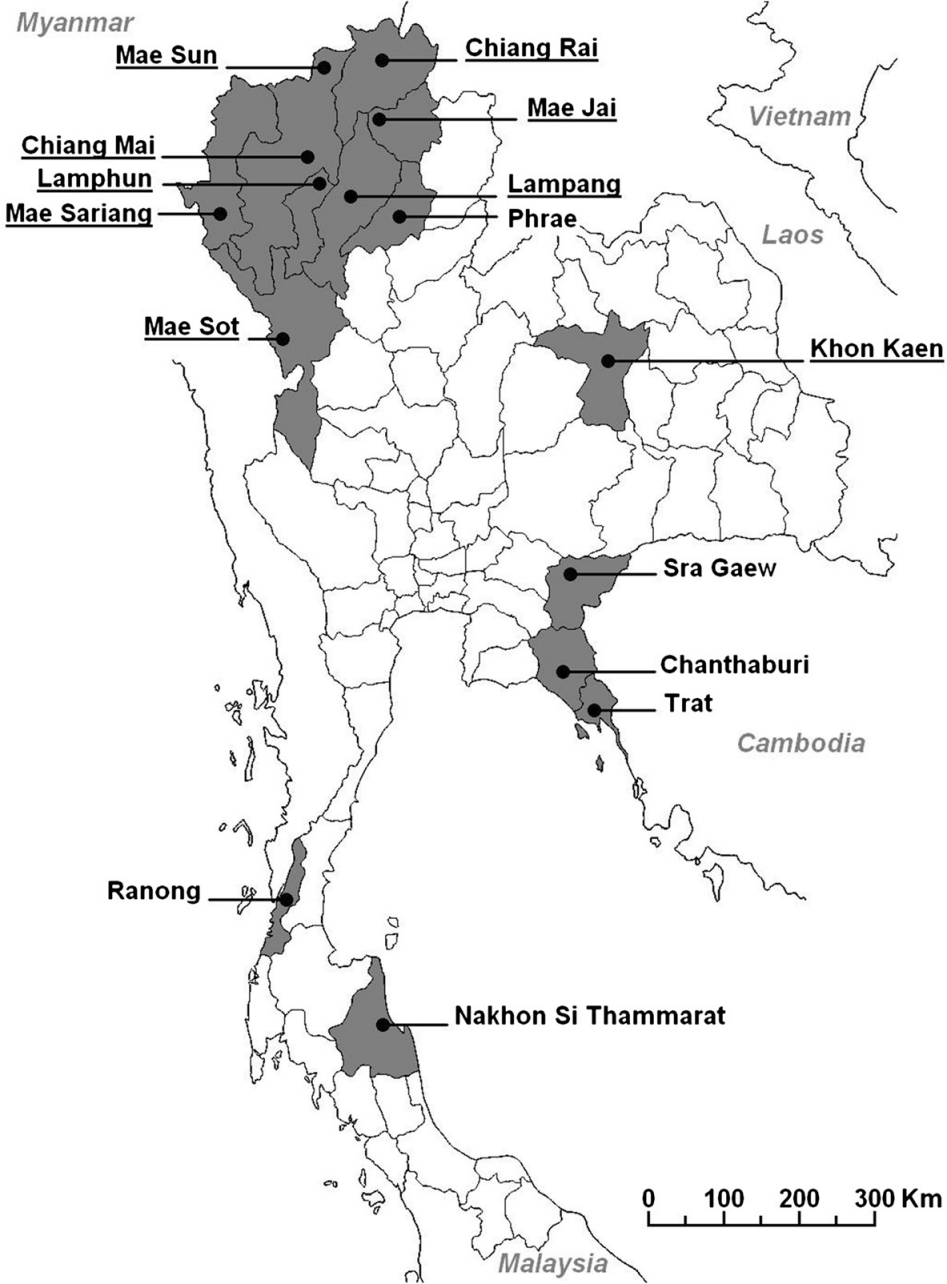
**Larval collection map.** Map showing locations of *Aedes aegypti* larval collections in Thailand. Underlined location names indicate sites from which collected larvae were reared to adulthood and tested with deltamethrin. Larvae from other provinces were killed and stored in absolute ethanol. No insecticide testing was performed on those samples.

Larvae and pupae were collected from artificial containers situated within domestic and peridomestic areas from selected urban locales. Sampled locations consisted of detached housing, temples, schools, as well as higher-density residential areas including alleys and walkways near apartments. Larvae were collected from a total of 14 provinces from throughout Thailand (Figure 1). Additionally, a number of larvae collected from Lahore, Pakistan were given to our department for testing. Larvae obtained from 6 Thai provinces, as well as those from Pakistan, were submitted preserved in absolute ethanol. Those collected from the other provinces were brought back to our insectary and reared under standard insectary conditions. Larvae were maintained on a diet of finely ground fish food until pupation, at which time pupae were separated according to sex. Upon emergence, female mosquitoes were maintained in small holding containers and provided a 10% w/v solution of sucrose absorbed onto a ball of cotton. To prevent injury or overcrowding, approximately 25–30 mosquitoes were kept in each holding container. Females were maintained under a 12:12 light: dark cycle under 80% RH. No blood meals were provided. No research involving vertebrates or regulated invertebrates was conducted during this study, therefore approval by the Chiang Mai University ethics committee was not required.

### Deltamethrin bioassays

One to three day old female mosquitoes were used for deltamethrin susceptibility testing. Test procedures followed standard WHO protocols [18]. At least 100 females obtained from each location were used for testing, if available. This provided four replicates of 25 mosquitoes. Following the procedure, each replicate group was placed into a holding tube lined with filter paper and initially observed to determine if injured or otherwise unsuitable mosquitoes were present. Thereafter they were transferred into exposure tubes, each lined with 0.05% deltamethrin-impregnated papers (WHO, Malaysia). A control group, not exposed to insecticide, but transferred to another holding tube, was also used. Insecticide exposure lasted one hour. Thereafter, knockdown individuals were scored. Following exposure, the mosquitoes were reintroduced into their respective holding tubes and again provided a 10% w/v sucrose solution. After 24 hours dead mosquitoes, as well as those alive but incapable of coordinated movement, were scored as susceptible (S). Remaining survivors were scored as resistant (R). All samples were subsequently stored in absolute ethanol.

### V1016G AS-PCR assay development and usage

**Figure 2:**
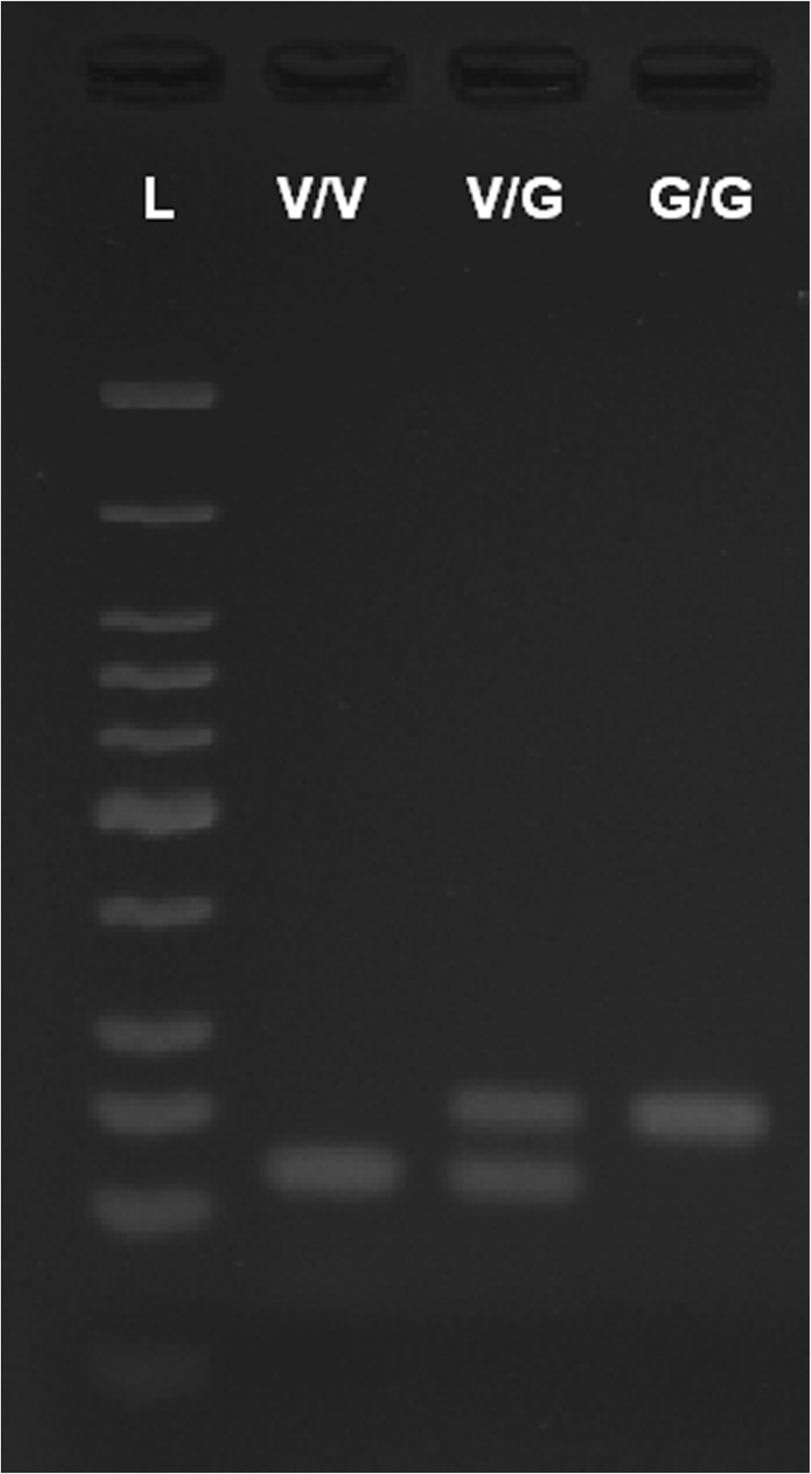
**Gel electrophoresis results.** Each of the three genotypes is shown from left to right: wild-type homozygous (V/V), heterozygous (V/G) and mutant homozygous (G/G). The lane to the far left contains low-molecular weight DNA ladder (L).

From each test of 100 mosquitoes, a total of 40 were processed for genotyping, 20 each from among susceptible and resistant mosquitoes, if available. Genomic DNA was obtained by using DNAzol® DNA extraction reagent (Invitrogen, USA). Extraction was performed according to the manufacturer’s instructions, except that homogenized mosquito samples were incubated for 24 hours prior to further processing. DNA concentration was measured using a Nanodrop 2000 spectrophotometer (Thermo Scientific, USA) at 260 nm. Stock solutions were prepared at a concentration of 25 ng/μl and used for AS-PCR genotyping. For our study, we sought an AS-PCR assay which would utilize a standard PCR thermal cycler and the products of which could be visualized by gel electrophoresis. Our assay utilizes allele-specific primers previously developed for an assay optimized for RT-PCR use [15]. Each reaction was performed in a 10 μl volume consisting of 1.5 mM MgCl_2_, 1x PCR buffer (Invitrogen, USA), 0.25 μM forward primer (5′-ACCGACAAATTGTTTCCC-3′), 0.125 μM each reverse primer specific for either Gly (5′-GCGGGCAGGGCGGCGGGGGCGGGGCCAGCAAGGCTAAGAAAAGGTTAACTC-3′) or Val (5′-GCGGGCAGCAAGGCTAAGAAAAGGTTAATTA-3′), 200 μM dNTP mixture (New England Biolabs, USA), 0.2 units Taq polymerase (Invitrogen, USA) and 25 ng genomic DNA. The thermal cycling condition begins with an initial DNA denaturation step for two minutes at 94°C, followed by 35 cycles of 30 sec at 94°C (denature), 30 sec at 55°C (anneal), and 30 sec at 72°C (extension). This is then followed by two minutes at 72°C for final extension. Since the primers used had GC-rich tails of varying lengths, amplified products could be differentiated by size (60 bp for Val, and 80 bp for Gly) (Figure 2). PCR amplification products were loaded onto a 4% agarose gel and run for 50 min at 100 V in TBE buffer.

### F1534C AS-PCR genotyping

In order to determine the contribution of both the V1016G and the F1534C mutations towards deltamethrin resistance, a number of samples including larvae, and susceptible and resistant adults were tested for the F1534C mutation which is known to confer resistance to permethrin. Testing for this mutation was conducted after testing for V1016G. The AS-PCR assay follows the protocol of one of our previous studies [8]. Each reaction was performed in a 10 μl volume with final concentrations of 1.5 mM MgCl_2_, 1x PCR buffer, 0.5 μM Phe forward primer (5′-GCGGGCTCTACTTTGTGTTCTTCATCATATT-3′), 0.165 μM Cys forward primer (5′-GCGGGCAGGGCGGCGGGGGCGGGGCCTCTACTTTGTGTTCTTCATCATGTG-3′), 0.5 μM common reverse primer(5′-TCTGCTCGTTGAAGTTGTCGAT-3′), 200 μM dNTP mix, 0.2 units Platinum *Taq* DNA polymerase, and 25 ng template DNA. Reactions were run at 95°C for 2 min initial activation stage and followed by 35 cycles of 95°C for 30 sec, 60°C for 30 sec, and 72°C for 30 sec, in turn followed by a final extension at 72°C for 2 min. PCR products were loaded onto 3% agarose gels and electrophoresis was conducted ay 100 V for 45 min.

### DNA sequencing

To validate the results obtained from our AS-PCR test specific for the V1016G mutation, we sequenced some of the genotyped samples in order to determine the accuracy of the assay. We obtained 90 previously tested samples, 30 from each genotype, and used these samples for sequencing. We began by amplifying a fragment of domain II in the sodium channel gene that encompasses the V1016G mutation. The method has been described previously [8]. Each reaction was performed in a 20 μl reaction volume. Reagents are added to final concentrations of 1.5 mM MgCl_2_, 1x PCR buffer, and 0.5 μM each of forward (5′-GGTGGAACTTCAC-CGACTTC-3′) and reverse (5′-GGACGCAATCTGGCTTGTTA-3′) primers, 200 μM dNTP mix, and 0.4 units of Platinum *Taq* DNA polymerase. PCR amplification begins with 2 min at 95°C, followed by 35 cycles of 95°C for 30 s, 63°C for 30 s, 72°C for 30 s, and a final extension at 72°C for 2 min. Amplified products were purified using ExoStar DNA purification reagent (GE Illumina, USA). Purified samples were then sent to Macrogen, Inc. (Seoul, Korea) for sequencing. Sequencing reactions were performed on an ABI 3730XL DNA analyzer (Applied Biosystems Inc., USA). Resultant data were analyzed using Geneious software, version 5.3.6 (Biomatters Ltd., UK). A set of sequenced DNA samples representing each of the three genotypes was serially diluted from stock concentration and tested to determine the detection limit for the assay.

### Statistical analysis

Pearson’s *X*^2^ test was used to compare the genotype and allele frequencies between susceptible and resistant mosquito groups, as well as to compare differences between the combined adult samples and larvae. Fisher’s exact test was used to compare allele frequencies of dead and surviving mosquitoes at each sampled location. All tests and calculations were performed in R 2.15.1 [19].

## Results

### Deltamethrin bioassays and adult genotyping

A total of 1465 female *Ae. aegypti* mosquitoes collected from urban areas in 8 provinces were tested for deltamethrin susceptibility and resistance (Table 1). Overall susceptibility was 77.6%, with mortality rates varying widely from 50.0 % to 89.9%. Samples obtained from Chiang Rai, Mae Hong Son, Phayao Provinces show varying degrees of incipient resistance (80-97% mortality). There was no mortality among control mosquitoes. Genotype and allele frequencies were determined from 451 susceptible and 301 resistant mosquitoes selected at random. Genotype frequencies between resistant and susceptible mosquitoes were significantly different (*X*^2^ = 101.24, df = 2, P < 0.0001). The frequencies of the 1016G allele were significantly different (*X*^2^ = 95.19, df = 1, P < 0.0001) between dead and surviving mosquitoes as well (0.198 and 0.432, respectively).

**Table 1:** Deltamethrin bioassay and AS-PCR results

**Province**	**Location**	**Total**	**Mortality**	**Status**	**n**	**Total**	**Genotype**			**G Allele**	**G Allele**	**Fisher's exact test**
		**Tested**	**(%)**			**PCR**	**V/V**	**V/G**	**G/G**		**95% CI**	**P value (α = 0.05)**
Chiang Rai	Chiang Rai City	120	82.5	R	21	21	10	10	1	0.286	[0.172, 0.436]	0.00684
				S	99	20	18	2	0	0.050	[0.138, 0.165]	
Chiang Mai	Mae Sun	36	50.0	R	18	17	1	11	5	0.618	[0.450, 0.761]	0.02803
				S	18	17	6	11	0	0.324	[0.191, 0.492]	
Phayao	Mae Jai	109	85.3	R	16	16	15	1	0	0.031	[0.055, 0.157]	0.45710
				S	93	19	19	0	0	0.000	[0.000, 0.918]	
Chiang Mai	Chiang Mai City	538	75.7	R	131	126	16	72	38	0.587	[0.526, 0.646]	0.00000
				S	407	255	128	120	7	0.263	[0.226, 0.303]	
Lamphun	Lamphun City	99	66.7	R	33	20	11	9	0	0.225	[0.123, 0.375]	0.04763
				S	66	20	18	2	0	0.050	[0.014, 0.165]	
Lampang	Lampang City	100	79.0	R	21	20	15	5	0	0.125	[0.055, 0.261]	0.43150
				S	79	20	18	2	0	0.050	[0.014, 0.165]	
Mae Hong Son	Mae Sariang	99	89.9	R	10	10	0	9	1	0.550	[0.342, 0.742]	0.04266
				S	89	20	10	10	0	0.250	[0.142, 0.402]	
Tak	Mae Sot	159	80.5	R	31	31	19	12	0	0.194	[0.114, 0.309]	0.00448
				S	128	40	37	3	0	0.038	[0.013, 0.105]	
Khon Kaen	Khon Kaen City	205	77.1	R	47	40	10	19	11	0.513	[0.405, 0.619]	0.00000
				S	158	40	25	15	0	0.188	[0.117, 0.287]	
Total		1465	77.6	R	328	301	97	148	56	0.432	[0.393, 0.472]	
				S	1137	451	279	165	7	0.198	[0.174, 0.226]	

A sample of the total number of deltamethrin tested mosquitoes (n) was used for PCR testing. Samples of susceptible (S) and resistant (R) mosquitoes were genotyped and mutant allele (G allele) frequencies calculated. Fisher’s exact test was used to test G allele frequency differences between S and R mosquitoes from each location. For *X*^2^ results on the overall population genotype and allele frequencies, see results section. Locations are listed in order from north to south.

**Figure 3:**
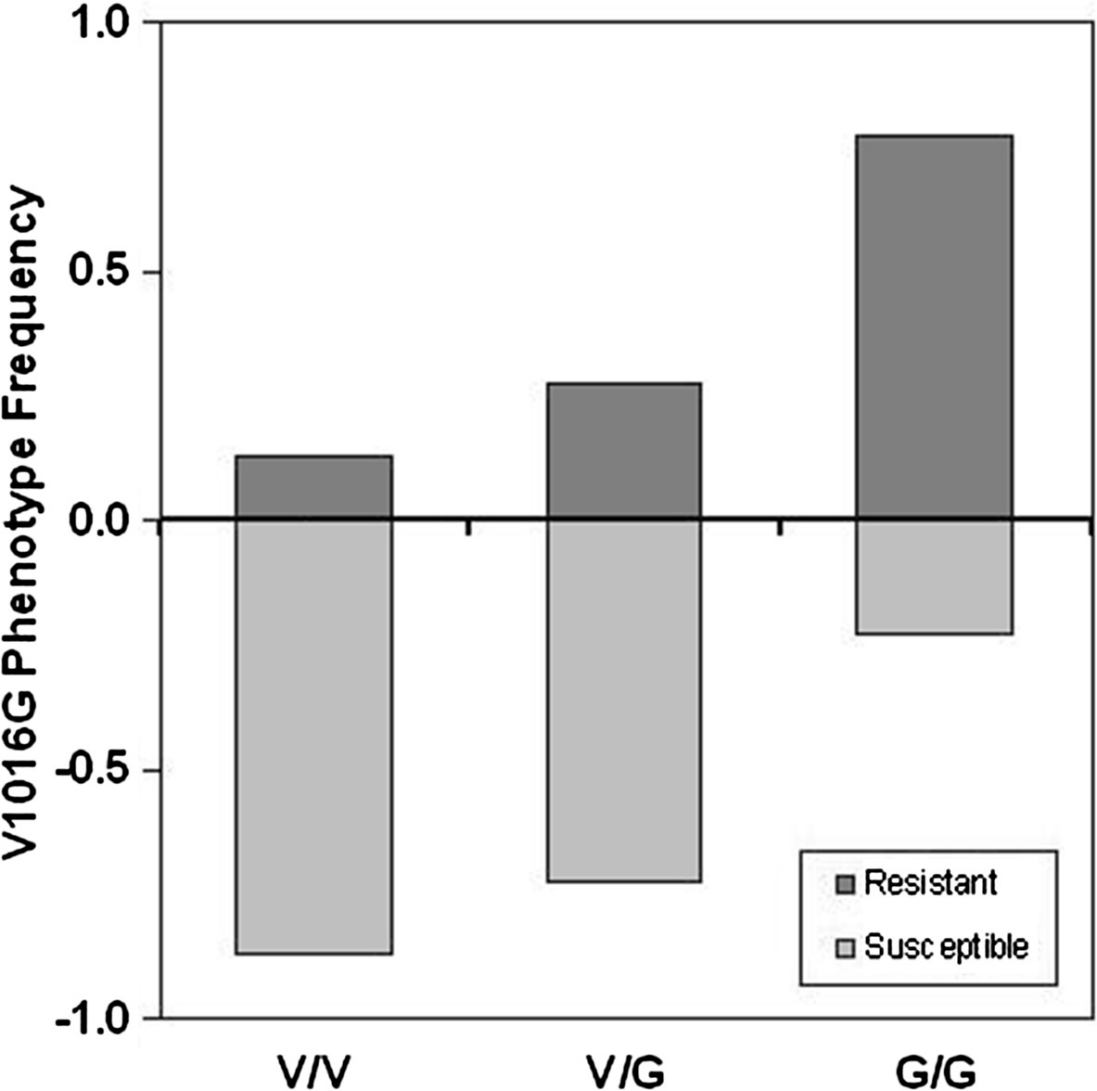
**Correlation between V1016G genotypes and the deltamethrin resistance phenotype.** The survival of individual genotypes was compared to determine the contribution of the V1016G mutation to deltamethrin resistance.

Fisher’s exact test was used to compare differences in 1016G allele frequencies between susceptible and resistant mosquitoes from each location. In most locations, the allele frequencies were highly significantly different between dead and surviving individuals; however, in those samples obtained from Lampang City (Lampang) and Mae Jai (Phayao), the differences in allele frequencies between susceptible and resistant groups were not significant (Table 1). These populations also had the lowest total mutant allele frequencies among those sampled (0.088 and 0.015, respectively). Furthermore we found that there was very little correlation when comparing 1016G frequencies directly against deltamethrin resistance at all locations (data not shown). We instead looked at the resistance phenotype of each genotype under selection with deltamethrin (Figure 3). Mutant homozygous mosquitoes survive at much higher rates. By multiplying the total number of insecticide tested mosquitoes against the genotype frequencies of the AS-PCR tested samples, we deduced the absolute phenotypic frequencies. Mutant homozygous mosquitoes had a resistance phenotype frequency of 0.772, whereas heterozygous and wild-type homozygous individuals show much reduced rates (0.279 and 0.131, respectively).

### Larval genotyping

In addition to our adult testing, larvae from six provinces were also genotyped. (Figure 1, non-underlined location names). Samples represent areas from the north (Phrae), south (Ranong and Nakhon Si Thammarat) and the southeast of Thailand (Chanthaburi, Sra Gaew and Trat). A total of 128 larvae were genotyped. The frequency of the 1016G allele was 0.305 (Table 2). The frequency of mutant homozygous individuals was also found to be higher among larvae than that seen from the adult data (0.125 and 0.084, respectively), however this difference was not significant (*X*^2^ = 1.85, df = 1, P = 0.174).

**Table 2:** Larval genotyping results

**Province**	**Location**	**PCR**	**V/V**	**V/G**	**G/G**	**G Allele**	**G Allele 95% CI**
Phrae	City	18	15	3	0	0.083	[0.029, 0.218]
Sra Gaew	City	22	17	5	0	0.114	[0.049, 0.239]
Chanthaburi	City	12	4	7	1	0.375	[0.212, 0.573]
Trat	City	16	8	7	1	0.281	[0.156, 0.454]
Ranong	City	30	9	11	10	0.517	[0.393, 0.638]
Nakhon Si Thammarat	City	30	13	13	4	0.350	[0.242, 0.476]
Total		128	66	46	16	0.305	[0.252, 0.364]

Locations refer to capitol cities named after their respective provinces with the same name. Due to the collection method, no insecticide testing could be performed. Locations are listed from north to south.

### AS-PCR for the F1534C mutation

In order to determine the role that another common *kdr* mutation might play in contributing to deltamethrin resistance, we took 170 individuals previously genotyped for the V1016G mutation and tested them for the presence of the F1534C mutation (Table 3). A total of 62 larvae, as well as 47 susceptible and 61 resistant adult females, with representatives from all provinces, were tested. All three genotypes at position 1016 were represented (74 V/V, 66 V/G and 30 G/G). In our testing we found no individuals that expressed the wild-type at both positions simultaneously, nor were any found that expressed both mutations (double homozygous mutant). Double heterozygous samples were common; however, no samples expressed the homozygous type of one mutation combined with a heterozygous type for the other. Among the samples tested were larvae obtained from Lahore, Pakistan. Initial testing of 50 such larvae indicated they were all wild homozygous (V/V) at position 1016 (data not shown); but upon further testing of 23 of these samples, we found that all were mutant homozygous (C/C) at position 1534 (Table 3).

**Table 3:** AS-PCR genotyping for F1534C

		**Larvae** **n = 62**				**Susceptible** **n = 47**				**Resistant** **n = 61**		
V1016G		V/V	V/G	G/G		V/V	V/G	G/G		V/V	V/G	G/G
	F/F	0	0	10	F/F	0	0	7	F/F	0	0	13
F1534C	F/C	0	10	0	F/C	0	20	0	F/C	0	36	0
	C/C	42	0	0	C/C	20	0	0	C/C	12	0	0

Samples of larval and adult mosquitoes previously genotyped by V1016G AS-PCR were selected for testing to detect the presence of the F1534C mutation. A total of 170 samples were tested.

### Sequencing of domain II of the sodium channel gene

Ninety samples, each distributed evenly among the different genotypes (30 each), were sequenced. All sequenced samples were in agreement with results previously obtained by AS-PCR testing. Sequencing also revealed the presence of the serine to phenylalanine transversion at position 989 within domain II (S989P) alongside V1016G. A single heterozygous resistant female from Chiang Rai also expressed the homozygous I1011V mutation. From three of these sequenced samples, a set of serial dilutions were made for each genotype and tested in order to determine the limit of detection. Homozygous samples of either genotype could be detected at 0.5 ng/μl, however, reliable detection of heterozygous individuals occurred down to 1.0 ng/μl of genomic DNA. Thus a concentration of 1.0 ng/μl represents the limit of detection for this assay.

## Discussion

In this study, we have successfully developed a simple AS-PCR technique to detect the V1016G mutation in Thai populations of *Ae. aegypti*. The high level of agreement between this assay and sequenced samples should encourage the use of this assay in order to determine the full extent of this mutation throughout Southeast Asia and elsewhere. This mutation is evidently widespread throughout Thailand, although individuals homozygous for the 1016G allele are still relatively uncommon. The mutant allele was found, to some extent, in material from all locations. Unfortunately, all of these populations display some degree of resistance to deltamethrin, with an overall 77.6% mortality rate. This resistance level is considered to be an underestimate due to our use of 0.05% deltamethrin paper which is at a higher concentration than the discriminating dose (0.025%) for adult *Ae. aegypti* recommended by WHO [18]. Deltamethrin resistance rates and V1016G allele frequencies varied widely between locations and no strong correlation could be seen between deltamethrin survival and mutant allele frequencies. However, most homozygous mutant females were resistant and most wild-type homozygous were susceptible, whereas heterozygous mosquitoes displayed intermediate resistance to deltamethrin. This may be explained because the 1016G allele is recessive for the *kdr* characteristic [8,9]. The survival of approximately a third of the heterozygous mosquitoes also likely indicates that the 1016G mutation is not the only mechanism involved and that other *kdr* or enzymatic mechanisms may confer cross resistance or enhance resistance.

Interestingly, no homozygous 1016G mutants were ever found that also expressed the homozygous form of the 1534C mutation, regardless of deltamethrin exposure status. Of 170 mosquitoes checked, these double mutants were never found, either in resistant or susceptible insects. Similarly, no double wild-type mosquitoes were found, indicating that in Thailand at least, *Ae. aegypti* harbor either the 1016G mutation, or the more common and widespread 1534C mutation. This is not to say double wild-type mosquitoes do not exist, but such specimens are likely quite rare in the wild as a result of both extensive and intensive pyrethroid usage throughout Thailand. This indicates that the ability to control *Ae. aegypti*, especially using pyrethroids such as permethrin, has been severely compromised. An exception to this is the pyrethroid-susceptible PMD strain that has been maintained in our insectary and was originally collected from a rural area of Chiang Mai province [20]. This strain harbors neither of the aforementioned *kdr* mutations [21]. Currently the 1016G mutation has thus far only been found within Southeast Asia including Bhutan. Our testing of *Ae. aegypti* larvae from Lahore, Pakistan revealed that the mutation was not present there. However, upon further examination, all larvae were homozygous for the 1534C mutation. This is therefore the first report of that mutation in Pakistan and likely indicates a population that is highly resistant to type I pyrethroid insecticides.

The 1534C mutation is far more common than the 1016G mutation. An earlier study revealed an allele frequency of 0.77 for the 1534C mutation in Thailand [8]. Mosquitoes with the homozygous 1534C mutation are generally susceptible to deltamethrin. The PMD-R strain maintained in our insectary is homozygous 1534C and exhibits 100% mortality after 1 h exposure to 0.05% deltamethrin paper (unpublished data). Conversely, specimens homozygous for the 1016G allele would likely be protected. The finding that these two mutations are apparently never found together in the same individual, other than in heterozygous forms, contrasts with results found in other regions. In the Cayman Islands, for example, a number of mosquitoes were found that were homozygous for two mutations, F1534C and V1016I [9]. A small number of samples were also wild-type homozygous at both positions. In Vietnam, many larvae were found to lack both mutations, and only two larvae were found to be 1016G heterozygous [10].

One limitation of this study is that we did not conduct insecticide testing in combination with the synergist PBO which suppresses the activity of detoxifying enzymes such as P450s and non-specific esterases, and thus may modify or reduce metabolic resistance to pyrethroids. Although the V1016G mutation is associated with deltamethrin resistance, as found in previous studies [12,13], and further corroborated in this study, it may not explain the resistance phenotype completely [22]. Clearly, individuals expressing the homozygous form of the mutation have much higher chances of survival under pyrethroid selection, but it should be noted that a greater number of homozygous wild-type individuals survived exposure (56 G/G and 97 V/V, Table 1), albeit at much lower frequencies than their mutant counterparts. There are likely a number of metabolic resistance mechanisms at work. It is also possible that these ‘wild-type’ mosquitoes harbor the homozygous 1534C mutation, as seen by our testing (Table 3). And although this mutation is correlated with resistance to type I pyrethroids, it does not contribute to resistance to type II pyrethroids, as indicated by the low numbers of 1016 V homozygous (and thus 1534C mutant homozygous) individuals that survived. This has been previously confirmed by the insertion of the 1534C equivalent mutation into the VGSC gene of the cockroach [23]. The channel remained sensitive to the action of deltamethrin, yet showed decreased sensitivity to permethrin.

In one study, low levels of deltamethrin resistance were found among *Ae. aegypti* populations sampled in central Thailand [24]. Sequencing of the partial sodium channel gene encoding segment domain II indicated that the 1016G mutation was not present, although two other uncharacterized polymorphisms were discovered. It was also determined that mixed function oxidases were elevated in all field collected material relative to a susceptible strain. The survival of such wild-type individuals after deltamethrin exposure is therefore likely due to metabolic mechanisms and warrants further investigation. Our previous study revealed that besides the F1534C mutation, oxidative enzyme systems also play a role in pyrethroid resistance in *Ae. aegypti* in Thailand [17].

From this study alone we cannot determine the contributions of metabolic mechanisms or other *kdr* mutations towards deltamethrin resistance. In many of our sampling locations we found that there are highly significant differences between the allele frequencies of susceptible and resistant mosquito groups. This provides strong evidence that the mutation contributes to deltamethrin resistance. However, in two populations, one of which displays incipient resistance (Mae Jai, 85.3% mortality) while the other appears resistant (Lampang City, 79.0% mortality), there were no significant differences between the mutant allele frequencies of susceptible and resistant groups (Fisher’s exact test, P = 0.4517 and P = 0.4315, respectively). These two populations also had the lowest 1016G allele frequencies among all sampled groups. There is the possibility that other *kdr* mutations are involved in deltamethrin resistance in Thailand. The V1016I mutation, for example, is more commonly found in Central and South America, and has been correlated to resistance to deltamethrin [25]. Nevertheless, both the V1016I and I1011V mutations were recently found in a deltamethrin-resistant strain from Vietnam [26]. The V1016I mutation has not been detected among Thai populations to date, however, I1011V has previously been found throughout Thailand at an allele frequency of 0.14 [11]. Based on our sequencing data, only one deltamethrin-resistant V1016G heterozygote was found to have the homozygous form of the I1011V mutation. At this time, little is known about the contribution of this polymorphism to insecticide resistance, and it has yet to be confirmed to reduce sodium-channel sensitivity to pyrethroids in *Xenopus* oocytes [27].

A similar mutation at the same position, I1011M, known from Latin American *Ae. aegypti* populations, was significantly associated with resistance to cypermethrin in a population from Brazil [28]. Mutations at this location may be important in conferring resistance to various pyrethroids and future studies should address this issue. Furthermore, the 1016G mutation is usually found with a 989P mutation as well [12]. Yet not all strains expressing the 1016G mutation simultaneously express 989P. A previously mentioned strain from Taiwan lacks 989P, but harbors a D1763Y polymorphism instead [14]. The 989P substitution was also lacking in some Thai and Indonesian strains that were homozygous for 1016G [13]. However, this mutation was apparent in our samples. More detailed studies are needed to determine what other polymorphisms may accompany this mutation and how they may contribute to the resistance phenotype. The fact that several different *kdr* mutations can be found within Southeast Asia underscores the need to further develop simple AS-PCR assays, as well as the need for multiplex PCR reactions to efficiently screen for multiple mutant alleles within a population.

## Conclusions

The assay we used proved to be highly reliable and this should aid future studies aimed at further determining the extent of this *kdr* mutation, particularly within Southeast Asia. The presence of the 1016G mutation was associated with resistance to deltamethrin and was found to be common among populations of *Ae. aegypti* throughout Thailand. No mosquitoes from the Thai populations sampled have yet been found to harbor both the 1016G and 1534C mutations. Wild type individuals without any *kdr* mutations are now probably very rare in Thailand. This is also the first report of the 1534C mutation in Pakistan and increases the known range of that mutation. The 1016G mutation appears to be confined to Southeast Asia at the present time.

## Competing interests

The authors declare that they have no competing interests.

## Authors’ contributions

NL, WC and PS conceived the study. AD and NL supervised the study. SAS and SP collected and tested mosquitoes. SAS, SP and JY designed and performed lab experiments. SAS, SP and JY analyzed the data. SAS, JY and PS interpreted the results. SAS wrote the draft manuscript. All authors read and approved the final manuscript.
